# An extensive phenotypic characterization of the hTNFα transgenic mice

**DOI:** 10.1186/1472-6793-7-13

**Published:** 2007-12-10

**Authors:** Michael D Hayward, Beverly K Jones, Arman Saparov, Heather S Hain, Anne-Cecile Trillat, Michelle M Bunzel, Aaron Corona, Bifang Li-Wang, Bryan Strenkowski, Caroline Giordano, Hai Shen, Emily Arcamone, Jeffrey Weidlick, Maria Vilensky, Marina Tugusheva, Roland H Felkner, William Campbell, Yu Rao, David S Grass, Olesia Buiakova

**Affiliations:** 1 Caliper Discovery Alliances & Services (Xenogen Biosciences), 5 Cedar Brook Drive, Cranbury, NJ 08512, USA

## Abstract

**Background:**

Tumor necrosis factor alpha (TNFα) is implicated in a wide variety of pathological and physiological processes, including chronic inflammatory conditions, coronary artery disease, diabetes, obesity, and cachexia. Transgenic mice expressing human TNFα (hTNFα) have previously been described as a model for progressive rheumatoid arthritis. In this report, we describe extensive characterization of an hTNFα transgenic mouse line.

**Results:**

In addition to arthritis, these hTNFα transgenic mice demonstrated major alterations in body composition, metabolic rate, leptin levels, response to a high-fat diet, bone mineral density and content, impaired fertility and male sexual function. Many phenotypes displayed an earlier onset and a higher degree of severity in males, pointing towards a significant degree of sexual dimorphism in response to deregulated expression of TNFα.

**Conclusion:**

These results highlight the potential usefulness of this transgenic model as a resource for studying the progressive effects of constitutively expressed low levels of circulating TNFα, a condition mimicking that observed in a number of human pathological conditions.

## Background

Increased production of the proinflammatory cytokine TNFα has been implicated in a number of human diseases involving inflammation such as autoimmune disorders, vascular disease and a number of cancers [1]. Most notably, aberrant expression of the TNFα gene is associated with rheumatoid arthritis (RA) in both humans and animal models [2-5]. In addition to the inflammatory component of RA, rheumatoid cachexia is characterized by reduced body weight, increased metabolic rate and restricted motor activity and also correlates with increased circulating TNFα levels [6]. Interestingly, TNFα is not only expressed in macrophages, lymphocytes, neutrophils, endothelial cells, keratinocytes and fibroblasts [7] but is also expressed in adipose tissue and is elevated in a number of experimental obesity models [8-11] and obese humans [12,13]. The pleiotropic actions of this cytokine have stimulated interest in animal models involving genetic manipulation of TNFα itself and components of its cellular signaling pathway.

Inactivation of the endogenous TNFα gene and its two cognate receptors have demonstrated its role in inflammation as well as obesity [14,15]. Transgenic mice expressing the TNFα gene under the control of various regulatory sequences have been used as models for RA as well as in vivo models to study wasting, ischemia, and lymphoid abnormalities [5,16-18]. Transgenic mice expressing a non-cleavable membrane bound TNFα have reduced adipose mass [19] and cardiac-specific overexpression of TNFα in mice results in cardiomyopathy [20]. The pleiotropic effects observed in genetic modifications of this cytokine *in vivo *therefore confirm the observations in humans and support the use of mutant mice as models for human diseases where a TNFα role has been described. A comprehensive phenotypic characterization of deregulated expression of TNFα would thus help inform investigators in their decision to use this specific animal model.

Our objective in this series of experiments was to determine the effects of deregulated hTNFα expression at low circulating levels on a broad range of murine physiological and behavioral measures. To achieve this goal, we comprehensively screened the hTNFα mice using sequential bioassays encompassing multiple physiological systems on the same groups of mice. A comprehensive phenotypic characterization of genetically-modified animals is limited by the time and expense in the selective breeding required to generate subjects for a large number of assays. To address this issue we have designed a serial phenotyping procedure used here that requires significantly fewer animals. By using such an approach, we were able to conduct a large number of tests in a relatively short period of time and used significantly fewer subjects than would be used in a study limited to experimentally naïve subjects. We have confidence that the results are reliable because we validated the order of these tests in wild-type animals by comparing data obtained by the serial protocol to data obtained in experimentally naïve subjects of comparable ages in pilot studies not included in this study. Because of the serial nature of our experimental approach, the results from the transgenic mice are relevant only to the age at which the specific assay was conducted and results could differ at different ages (e.g., due to the progressive-age related- deterioration of their health).

We found that in addition to serving as an improved model for progressive RA, these hTNFα transgenic mice exhibited numerous phenotypes including decreased activity, alterations in soft tissue and bone composition, impaired physical and metabolic responses to a high fat diet (HFD), increased food intake, markedly decreased leptin levels, significantly enhanced energy expenditure, impaired fertility and erectile dysfunction. The results from this study demonstrate that this transgenic line is a valuable model for the study of TNFα role in disease progression in multiple therapeutic areas including metabolism, obesity, bone homeostasis and male sexual health.

## Methods

### Animals

hTNFα transgenic (TG) and age- and sex-matched C57BL6/N wildtype (WT) mice were obtained from Taconic Farms (Hudson, NY, [21]). The hTNFα TG mice were generated using a construct that contains a 2.8 kb fragment of the human TNFα gene, including the entire coding region and promoter, fused to the human β-globin 3' untranslated region (UTR) that replaces the endogenous 3'UTR of the human TNFα gene [16]. This TG line was produced by pronuclear microinjection of B6SJL(F2) hybrid zygotes. The animals have been backcrossed for over 10 generations onto the C57BL6/N genetic background.

### Experimental Procedure

Mice were singly housed in a temperature- and humidity-controlled barrier facility. The mice were maintained on a 12 hr reverse dark/light cycle (lights go off at 11 AM). Animals had free access to food and water unless indicated otherwise in protocols for specific bioassays. All experiments were approved by the Xenogen Biosciences Institutional Animal Care and Use Committee.

The assays used in the initial screen are listed in Table 1. In the following studies mice were used in multiple assays unless otherwise noted. The order and corresponding ages of mice for each assay reported here is given in Table 2. Consecutive assays were always separated by a minimum of one week. The mice displayed good health based on gross observations. Assays for which significant differences between the hTNFα TG mice and WT controls were observed in the primary screen were repeated on experimentally naive animals of comparable ages to verify the initial results. The protocols of individual bioassays follow.

**Table 1:** List of bioassays used for characterization in the initial screen and result summary

**Therapeutic Field**	**Bioassays**	**Phenotype**
Cardiovascular, renal and bladder function	Bladder function (F)	NS
	Blood pressure/heart rate (M)	↑(BP)*
	Urine chemistry panel (F)	NS

CNS and Behavior	Irwin test (M,F)	↓***

Cross-therapeutic assays	Automated blood chemistry panel (M)	NS
	Body weight measurements (M,F)	NS
	Corticosterone (M)	↑*

Immunology and Inflammation	DTH (M)	NS
	FACS analysis (M)	↓*
	LPS challenge (M)	NS
	Monocyte infiltration (F)	↑*
	Pulmonary inflammation (F)	NS
	Wound healing (F)	NS

Metabolism and Body Composition	DEXA (F)	↓*
	Excised bone DEXA (F)	↓**
	Food intake (M,F)	NS
	Food intake on HFD (M)	↑***
	Leptin (M,F)	↓**
	Metabolic response to HFD (M)	↑**
	Physical response to HFD (M)	↓***
	Selected muscle weight (F)	↓**

Pain	Formalin pain assay (M)	NS
	Hot-plate assay (F)	NS

Sexual health	Histopathology of reproductive system (M)	NS
	Induced erection test (M)	↓*
	Male contact sexual behavior (M)	↓***
	Male fertility (M)	↓***
	Testosterone (M)	NS

The highest levels of significance among all measures measured in the assay are shown by asterisks: * P < 0.05, ** 0.001 < P < 0.01, *** P < 0.001 and arrows indicate measurements in the TG line increased or decreased from WT. No significant difference between genotypes (NS), males (M), females (F), delayed-type hypersensitivity (DTH), fluorescence-activated cell sorting (FACS), lipopolysaccharide (LPS), Dual energy X-ray analysis (DEXA) high fat diet (HFD).

**Table 2:** Age of mice and order of experiments

**Age (weeks)**	**Assay**	**Longitudinal Study**
8	LPS challenge	
15	Irwin	Body Weight (RC)
16	Food Intake	Body Weight (RC)
17	Male Fertility	Body Weight (RC)
18	Male Sexual Behavior	Body Weight (RC)
19	Male Sexual Behavior	Body Weight (RC)
20	Male Sexual Behavior Insulin	Body Weight (RC)
21	OGTT (pre-HFD)	Body Weight (RC)
22	DEXA(pre-HFD & Low Ca)	Body Weight (RC)
23	HFD Begins	Body Weight (RC)
		Body Weight (HFD)
24	Leptin	Body Weight (RC)
		Body Weight (HFD)
25		Body Weight (RC)
		Body Weight (HFD)
26		Body Weight (RC)
		Body Weight (HFD)
27	MicroCT OGTT (post-HFD)	Body Weight (RC)
	Insulin DEXA(post-Low Ca)	Body Weight (HFD)
28	DEXA (post HFD)	Body Weight (RC)
	CLAMS	Body Weight (HFD)
29	Insulin Tolerance Test	

### Lipopolysaccharide (LPS)-induced Inflammation

The mice were injected intraperitoneally (i.p.) with either 1 or 10 μg lipopolysaccharide (LPS, Sigma-Aldrich, Inc., St. Louis, MO) made up in 2 or 20 mg D-galactosamine (Sigma-Aldrich, Inc), respectively. Mice from the negative control group were injected with a phosphate buffered saline (PBS) vehicle alone. All mice were anesthetized with isofluorane 1.5 hours after injection, and blood was obtained from a retro-orbital vein. Serum was prepared by spinning clotted blood for 20 min at 4°C at 1200 × g. Human and mouse TNFα levels in the serum of the mice were detected by ELISA according to the manufacturer's protocol with a lower limit of detection in the mice assay of 23.4 pg/ml and 0.5 pg/ml in the human form (R&D Systems, Minneapolis, MN).

### Irwin Observational Battery

The mice were acclimated to the procedure room for at least 30 min prior to beginning the assay. The Irwin test battery is a modification of the original comprehensive observational assessment described by Irwin [18]. The behaviors were evaluated in one of four circumstances: a viewing jar, an arena, above the arena, or restrained. Forty-five endpoints were assessed and scored for each mouse. A description of the scoring for the measures given in Table 3 are as follows: Grip Strength (None = 0; Slight grip, semi-effective = 1; Moderate grip, effective = 2; Active grip, effective = 3; Unusually effective = 4). Wire maneuver (Active grip with hindlegs = 0; Difficulty to grasp with hindlegs = 1; Unable to grasp with hindlegs = 2; Unable to lift hindlegs, falls within seconds = 3 ; Falls immediately = 4). Tail elevation (Dragging = 0; Horizontally extended = 1; Elevated = 2).

**Table 3:** Phenotypes in the Irwin observational panel

**Observation**	**Genotype**	**Males**	**Females**
Body weight (g)	WT	31.5 ± 18.8	20.3 ± 0.9
	TG	25.5 ± 1.7*	22.2 ± 0.9
Body length (mm)	WT	92.8 ± 0.9	86.6 ± 0.5
	TG	89.4 ± 1.0*	87.8 ± 1.1
Grip strength (score 0–4)	WT	2.4 ± 0.2	1.0 ± 0
	TG	0.8 ± 0.2**	1.6 ± 0.2
Wire maneuver (score 0–4)	WT	1.4 ± 0.2	2.8 ± 0.5
	TG	4.0 ± 0***	2.0 ± 0.6
Tail elevation (score 0–2)	WT	2.0 ± 0	1.4 ± 0.2
	TG	1.2 ± 0.2*	1.2 ± 0.2

Values represent the mean ± SEM n = 5 mice for each sex/genotype combination. The level of significance is shown by asterisks: * *P *< 0.05, ** 0.001 <*P *< 0.01, *** *P *< 0.001. For scoring description see Methods section for Irwin observational panel.

Appropriate statistical tests were used to compare the TG mice to their WT counterparts (Fisher's exact t, Mann-Whitney U, unpaired t-tests).

### Food Intake on Regular Chow

The mice were singly housed in clean cages with one sheet of Iso-pad bedding (Harlan-Teklad, Indianapolis, IN) and a weigh boat containing dustless precision pellets (Bio-Serv, Frenchtown, NJ). They were habituated to this environment for 72 hours. At the end of the habituation period, the mice were placed in new cages containing Iso-pad bedding and 20 food pellets. The weights of both the food and the mice were recorded. The experimental phase lasted 72 hours at which point the remaining food was recovered and weighed. Food intake was expressed as mg food consumed/g body weight/24 hours. An unpaired t-test was used to analyze differences between genotypes.

### Leptin

Blood was collected via retro-orbital bleeds from fasted or freely fed mice. Serum was prepared by spinning clotted blood for 20 min at 4°C at 1200 × g and frozen at -20°C. The leptin ELISA was performed according to the manufacture's protocol (R&D Systems). An unpaired t-test was used to analyze differences due to genotype.

### Responses to HFD

The HFD challenge used the Western high fat/high carbohydrate diet (42.7 kcal % carbohydrate, 40.3 kcal % fat, 17 kcal % protein, D12079B, Research Diets, Inc., New Brunswick, NJ). The body weights of the mice were measured weekly and the following metabolic, anatomical and behavioral assays were conducted prior to and at the end of the HFD challenge.

### Whole Body Dual Energy X-ray Analysis (DEXA)

On the first day of the HFD the mice were weighed and their body composition was assessed by DEXA scan. The analysis was performed on a PIXImus2 X-ray unit (GE Lunar Corporation, Madison, WI) connected to a computer equipped with LUNAR PIXImus software. The head region was excluded from analysis. The following measures were directly made or calculated by the PIXImus2 software: bone mineral density, bone mineral content, bone area, tissue area, R soft tissue value (RST – ratio of attenuation of low energy and high energy beams in soft tissue), percent fat, and total tissue mass (TTM). Fat mass, percent of lean and lean mass were calculated manually as follows:

% Lean = 100 – % Fat

Lean Mass = TTM × (100 – % Fat)

Fat Mass = TTM × % Fat

An unpaired t-test was used to analyze differences between genotypes for each measure. At the end of the HFD period final body weights were taken and a post-HFD DEXA scan was also obtained.

### Food Intake on HFD

During week 4 of the HFD food intake measurements were performed while mice were singly housed in clean cages on standard corn cob bedding and weighed at the start of the experimental period, which lasted 72 hours. At the beginning of the assay, mice were given approximately 15 grams of pre-weighed high-fat food pellets. At the end of the experiment the remaining food was recovered and weighed. Food intake was expressed as the mg food consumed/g body weight/24 hours. An unpaired t-test was used to analyze differences due to genotype.

### MicroCT Analysis

During week 4 of the HFD the sizes of regional fat depots were determined by microCT analysis. Images of anesthetized mice were obtained using a commercially available microCT system (MicroCAT®, ImTek Inc. Oak Ridge, TN) with a high-resolution CCD/phosphor screen detector. Images were acquired with the X-ray biased at 40 kVp and 400 μA using a 0.5 mm aluminum filter. Each scan consisted of 196 individual projections with an exposure time of 320 ms/projection. Image reconstruction, whereby the 196 projections acquired in the scan were manipulated to produce two-dimensional cross sectional images of the mouse, was performed using the MicroCAT® Reconstruction, Visualization, and Analysis Software (ImTek Inc., Oak Ridge, TN). Slices were selected spanning the region between vertebral landmarks S4-S3 and L1–T13 using a 90° scout radiograph projection. Two-dimensional slices were reconstructed using a Shepp-Logan filtered back projection algorithm on a 256 × 256-pixel grid where the pixel size is 260 × 260 μm and the slice thickness is 1.6 mm. After reconstruction, the 2D-transaxial slice images were exported in *.bmp format for image analysis. Image analysis and volumetric fat calculations for renal/retroperitoneal, mesenteric, epididymal and inguinal depots were accomplished in 3D-Doctor (Able Software Corp., Lexington, MA). An unpaired t-test was used to analyze differences between mice of different genotypes for each parameter.

### Metabolic Responses to HFD

Mice fed the HFD for five weeks and mice maintained on regular low fat chow (rodent Purina diet #5053, Fisher Feeds, Bound Brook, NJ) were fasted overnight and then bled from the retro-orbital sinuses. The samples were left to coagulate and then centrifuged at 4°C to isolate the serum. The serum was immediately frozen and stored at -80°C. The frozen serum samples collected from all of the mice before and at the end of the HFD were defrosted and analyzed in parallel for serum insulin by ELISA (UltraSensitive Mouse Insulin ELISA; ALPCO, Salem, NH) according to the manufacturer recommendations. To assess glucose tolerance, mice maintained on regular chow or the same mice after feeding on the HFD for 5 weeks were fasted for 16 – 20 hrs prior to the oral glucose tolerance test (OGTT). The animals were weighed immediately before the test and then the baseline serum glucose level was measured in a drop of blood obtained from a cut at the tip of the tail. The glucose levels were measured by a Glucometer Elite (Bayer, Leverkusen, Germany). The mice then received 1 g/kg body weight of 100 mg/ml glucose solution (Sigma, St. Louis, MO) in sterile water delivered by oral gavage. At 30, 60 and 120 min after the gavage, glucose concentration was measured as described above. The insulin tolerance test (ITT) was performed on ad libitum fed males during the 7^th ^week of the HFD with human regular insulin at a dose of 75 U/kg injected i.p. Body weights, OGTT and ITT results were analyzed by two-way ANOVA. All other endpoints were also analyzed using an unpaired t-test to identify differences due to genotype.

### Comprehensive Laboratory Animal Monitoring System

Mice fed the HFD for six weeks and mice maintained on the regular chow diet were assessed for 48 hours in the Comprehensive Laboratory Animal Monitoring System (CLAMS, Columbus Instruments, Columbus, Ohio). Measurements included oxygen consumption (VO_2_), carbon dioxide production (VCO_2_), respiratory exchange ratio (RER, calculated), calculated heat production, total horizontal activity, ambulatory activity, non-ambulatory horizontal activity, vertical (rearing) activity and licking frequency. Results were averaged by photoperiod and analyzed by two-way ANOVA. For the heat production, oxygen consumption and carbon dioxide production endpoints, weight change was used as a covariate in the ANCOVA.

### Low Calcium Dietary Challenge

Following a baseline DEXA scan of bone composition, the mice were maintained on diet # D02040301 from Research Diets (New Brunswick, NJ) for a period of four weeks. This diet contains 0.01% calcium. After four weeks on the diet a second DEXA scan was performed. The percent change from baseline was calculated for each endpoint and an unpaired t-test was used to analyze differences between mice of different genotypes for each time point.

### Male Fertility

On the first day of testing, CD1 females were introduced to the experimental males before the beginning of the dark phase of the light/dark cycle and left with males for one week. Vaginal mating plugs were checked daily at the end of the light phase. On day 8 the pairs were separated. Females were individually housed and allowed to deliver litters. The number of litters and litter size were determined for males of each genotype.

### Male Contact Sexual Behavior

This assay was performed on the same male mice used in the Male Fertility assay. Prior to testing, the males were trained twice per week for two consecutive weeks. 8–10 Week old ovariectomized ICR female mice (Taconic Farms, Hudson, NY) implanted with a 2.5 mg 17-β-estradiol pellet (Innovative Research of America, Sarasota, FL) and injected with progesterone (Sigma-Aldrich, Inc) (500 μg in 0.2 ml of sesame oil 5 hours prior to the start of the experiment) were introduced to the cages of experimental males 1 hr after the onset of the dark phase for 4 hrs. After each training session females were observed for copulative plugs. Thirty minutes prior to the testing session the males were evaluated in the induced erection test as described below. Then the ovariectomized females supplemented with estrogen and progesterone were introduced to the male cages. The cages were placed in testing video cabinets (Noldus Information Technology, Wageningen, Netherlands) and sessions were digitally recorded for 45 min (Numeriscope™, Viewpoint, Champagne, France). The testing sessions were analyzed by a trained operator blind to the genotype using Observer software (Noldus Information Technology). The total number of mounts, intromissions and ejaculations were determined for every male. Latencies to the first mount and to the first intromission (from the beginning of the experiment) and latency to ejaculation from the first mount were also determined.

### Induced Erection Test

The testing was performed during the dark phase of the light cycle under red light. The male mice were held tightly in supine position. The penile sheath was retracted and gentle pressure was applied to the mouse abdomen for 10–15 s. The occurrence of penile erection was recorded. Erections were scored as present (1) or absent (0). Between group differences were analyzed by Chi-square test.

### Histolopathological Evaluation of the Reproductive Tissues

The reproductive tissues from two TG and two WT male mice were fixed in 4% formalin and sent without genotype identification to the Pathology Associates, a Charles River Company (Wilmington, MA) for histopathological evaluation. Testes were trimmed, embedded in glycol methacrylate (GMA), and stained with PAS/hematoxylin. The epididymides, prostates, seminal vesicles and penises were trimmed, embedded in paraffin, sectioned at approximately 5 microns, and stained with hematoxylin and eosin. Tissues were examined microscopically by a certified veterinary pathologist.

### Excised Bone DEXA Analysis

The animals were euthanized and the right femurs and the L1 – L6 region of the spine were excised from the bodies and scanned on PIXImus2 as described in the manufacturer's manual. The region of interest was adjusted to fit the excised bone only. For both excised femur and L1 – L6 spine, the following measurements were made: bone mineral density, bone mineral content, bone area, bone length and width. An unpaired t-test was used to analyze differences between mice of different genotypes for each measure.

### Staticstical Analysis

Statistical analysis was performed for each assay as indicated. Two-factor ANOVAs (with or without repeated measures) were performed using the SPSS statistical package (SPSS Inc., Chicago, IL). T-tests (paired or unpaired) were performed using Graphpad Prism (San Diego, CA) or Microsoft Excel. The data were assessed for outliers defined as two or more standard deviations from the mean and values exceeding these parameters were excluded.

## Results

### Characterization of Transgene Expression

**Figure 1:**
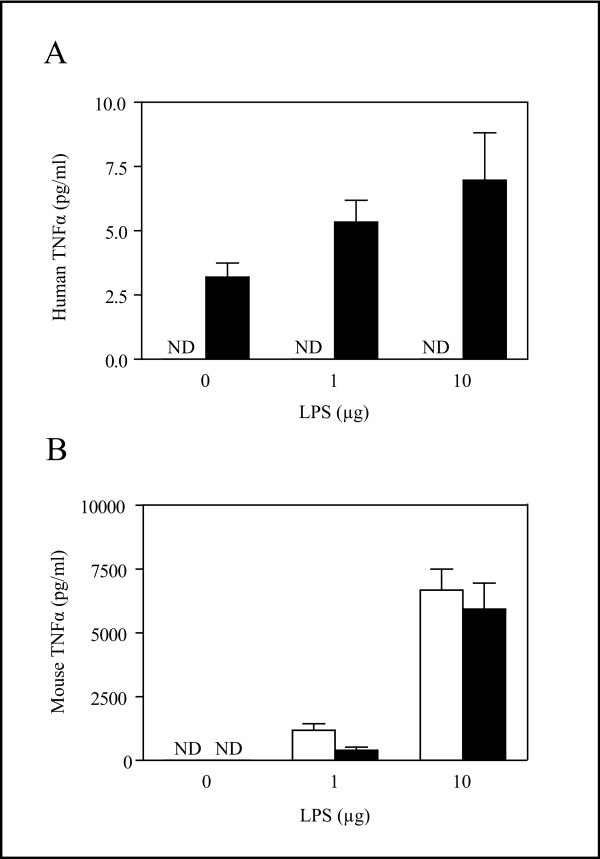
**Serum TNFα Levels After IP LPS Challenge**. LPS did not significantly alter expression of the transgene but dose-dependently increased endogenous TNFα in WT and TG mice similarly. WT (open bars) and TG mice (closed bars) were injected with the indicated doses of LPS (μg/mouse) as shown on the X-axis. (A) Human and (B) mouse TNFα levels were measured using ELISA assays. Data are presented as means ± SEM, n = 5 for each genotype and dose combination. ND – non-detectable.

To characterize the expression and regulation of the hTNFα transgene, we measured circulating hTNFα in unchallenged mice and tested it's inducibility relative to the endogenous mouse TNFα gene using increasing doses of LPS. Sera from 5 TG and 5 WT males were assayed with two ELISA systems specific for human or murine TNFα protein respectively, after *i.p*. injection with vehicle (PBS), 1 μg LPS or 10 μg LPS. In the TG mice, hTNFα protein was detectable at low levels in all treatment groups and did not significantly change following treatment with the two LPS doses (Figure 1A). As expected, mouse TNFα levels in TG and WT mice injected with PBS were undetectable but we observed a robust dose-dependent induction of the murine TNFα gene in both the TG and WT animals following LPS treatment (Figure 1B). Additionally, the hTNFα transgene appeared to have no impact on endogenous TNFα production since no effect of genotype was detected by two-factor ANOVA. In a separate experiment we compared hTNFα levels in untreated male and female TG mice with WT littermates as negative controls. Very low levels of circulating hTNFα, which were only detectable with a highly sensitive assay, were measured in the TG mice (males 5.83 ± 0.58 pg/ml; females 6.51 ± 0.48 pg/ml, n = 5 for each sex/genotype combination). hTNFα was undetectable in the WT mice.

### Irwin Observational Panel

The Irwin Observational Panel was performed on mice at 15 weeks of age. This test encompasses basic observations in four categories: physical characteristics, autonomic responses, behavioral responses, and neurological responses [22]. The male TG mice differed significantly from WT mice in 5 out of 45 observations (Table 3). Assessment of physical characteristics found that body weight and body length were decreased in male hTNFα mice. Results in three tests designed to measure neurological responses related to muscle tone differed between the male TG and WT mice: hTNFα TG males exhibited reduced grip strength, were unable to grasp a wire in the wire maneuver test and displayed less elevated tail positions than the WT males (Table 3). Interestingly, the female TG mice at this age did not differ from their WT counterparts in any observation (Table 3). The sexual dimorphism seen in the Irwin test suggests that despite possessing similar levels of circulating hTNFα, the female TNFα mice had a milder phenotype than age-matched males, which is consistent with our observation that they tend to manifest arthritic symptoms later than the male TG animals (data not shown).

### Energy Homeostasis on Regular Chow

#### Food Intake, Body Weight and Soft Tissue Composition

**Figure 2:**
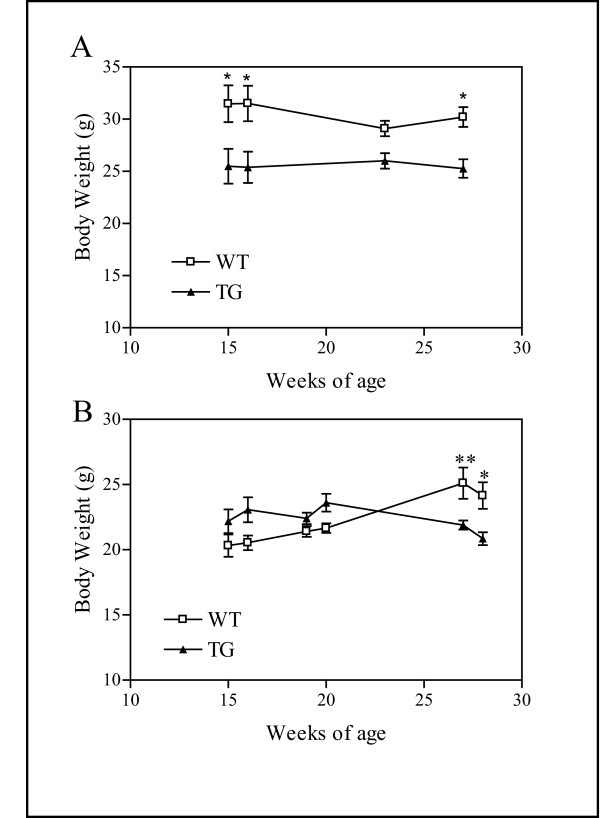
**Body Weights**. TG males were significantly lighter than WT males and the weight of TG females did not increase over time like WT females (A) Weights of male WT (open squares n = 4) and TG (black triangles n = 4) mice between 15 and 26 weeks of age. (B) Weights of female WT (open squares n = 6) and TG (black triangles n = 6) mice between 15 and 28 weeks of age. Results represent the mean ± SEM. The level of significance is shown by asterisks: * *P *< 0.05, ** 0.001 <*P *< 0.01, *** *P *< 0.001 in the post-hoc analysis.

Food intake was studied in 16 week old mice and the amount of food consumed per gram of body weight per 24 hours was calculated. There was no statistically significant difference in the amount of food consumed by the hTNFα TG mice as compared to the WT controls when both sexes were combined (WT: 105 ± 14 mg/gm BW/24 hrs vs TG: 99 ± 9 mg/gm BW/24 hrs) and no difference when results from males (WT: 77 ± 5 mg/gm BW/24 hrs vs TG: 88.2 ± 12 mg/gm BW/24 hrs) or females (WT:129 ± 20 mg/gm BW/24 hrs vs TG:110 ± 12 mg/gm BW/24 hrs) were analyzed separately (n = 5 for each sex/genotype combination). Similarly, no difference in total food intake was observed between genotypes when the total amount of food consumed per day was assessed without normalizing to body weight (data not shown). To establish a longitudinal body weight profile of the TG mice weights of 4 males and 6 females of each genotype maintained on regular chow were taken at four intervals between 15 and 28 weeks of age. Consistent with body weight results from the Irwin test we found that the TG males were significantly lighter than WT controls (ANOVA *F*_1,38 _= 33.73, *P *< 0.0001) over the 13 week period (Figure 2A). These data suggest a change in energy homeostasis since the differences in body weights between the TG and WT mice were not due to differences in food consumption. The body weights of females between 15 to 28 weeks of age did not significantly differ by genotype but the interaction between time and genotype was significant (ANOVA *F*_5,69 _= 6.74, *P *< 0.0001), indicating that WT females gained weight over the period measured but TG females did not (Figure 2B).

Consistent with the difference in body weights between the TG and WT males, DEXA analysis of 22-week-old TG male mice maintained on regular chow showed significant reductions in total tissue mass, total tissue area, fat mass, and lean mass (Table 4). Fat mass was 50% lower in the TG group and lean mass was reduced by 14%. The TG mice were relatively leaner than WTs as demonstrated by a significant increase in percent lean values. Overall, these results demonstrate that both fat mass and lean mass were decreased in the TG males, but that fat mass was decreased more than lean mass. In contrast, 22-week-old hTNFα females on regular chow did not exhibit significant differences in the relative proportions of fat and lean mass as determined by DEXA analysis relative to the WT controls (Table 4). Both the results of the soft tissue body composition analysis, and the later divergence in body weight in the females, suggest a sexual dimorphism in the response to similar levels of circulating hTNFα, consistent with the delayed onset of arthritis in females compared to males (data not shown).

**Table 4:** Soft tissue composition and body weight

**Parameter**	**Genotype**	**Females**	**Males**	
		
		***RC***	***RC***	***HFD***
Tissue area	WT	22.99 ± 0.30 (n = 7)	27.34 ± 0.59 (n = 8)	31.70 ± 0.67 (n = 8)
	TG	21.96 ± 0.51 (n = 5)	24.66 ± 0.33**(n = 7)	24.87 ± 0.49*** (n = 5)
Total tissue mass	WT	22.18 ± 0.57	32.00 ± 1.22	40.46 ± 1.48
	TG	20.92 ± 0.69	26.09 ± 0.53**	26.63 ± 0.97***
Fat mass	WT	2.47 ± 0.22	6.37 ± 0.8	15.33 ± 1.05
	TG	1.97 ± 0.12	3.20 ± 0.26**	5.45 ± 0.68***
Lean mass	WT	19.71 ± 0.36	25.63 ± 0.59	25.13 ± 0.47
	TG	18.95 ± 0.57	22.89 ± 0.35**	21.81 ± 0.35***
% fat	WT	11.15 ± 0.72	19.53 ± 1.67	37.57 ± 1.37
	TG	9.39 ± 0.28	12.20 ± 0.82**	20.08 ± 1.91***
% lean	WT	88.85 ± 0.72	80.47 ± 1.67	62.43 ± 1.37
	TG	90.61 ± 0.28	87.80 ± 0.82**	79.92 ± 1.91***
Body weight	WT	24.16 ± 1.02	32.19 ± 1.15	40.17 ± 1.46
	TG	22.01 ± 0.75	26.46 ± 0.50**	26.72 ± 0.91***

Values represent the mean ± SEM. RC is regular chow and HFD is high fat diet. The level of significance is shown by asterisks: * *P *< 0.05, ** 0.001 <*P *< 0.01, *** *P *< 0.001.

#### Circulating leptin

Leptin levels were measured in serum from 25 week-old fasted and 26 week old *ad libitum *fed males maintained on regular chow. Both fasted and fed blood leptin levels were significantly lower in TG males compared to WT animals (WT fasted: 2.663 ± 0.590 ng/ml [n = 7] vs. TG fasted: 0.019 ± 0.012 ng/ml [n = 7], *P *= 0.004; WT fed: 5.997 ± 0.758 ng/ml [n = 7] vs. TG fed: 1.015 ± 0.270 ng/ml [n = 6], *P *= 0.0004). Leptin levels usually correlate closely with the amount of white adipose tissue. However, in the hTNFα TG males leptin levels appeared disproportionately low as they were undetectable in fasted mice while in the fed state were reduced six-fold, but fat mass in the TG was reduced only two-fold. Twenty six week old TG females also showed a marked decrease in leptin levels compared to the WT controls; leptin levels in fasted WT females were 1.356 ± 0.385 ng/ml [n = 6] but were 0.144 ± 0.089 ng/ml [n = 6] in TG females (*P *= 0.028). These data demonstrate a striking dissociation between fat mass and leptin secretion since TG females had unchanged fat mass compared to WT but exhibited an almost ten-fold reduction in circulating leptin levels. Additionally, the food-intake measurements in mice fed regular diet did not detect any difference between genotypes despite significantly lower leptin levels. As leptin was measured nine to ten weeks after the food intake experiment we cannot rule out the possibility that the reduced serum leptin levels were a manifestation of a progressive decrease in body weight of the TG mice.

### Energy Homeostasis on HFD

#### Food intake, weight gain and soft tissue composition

**Figure 3:**
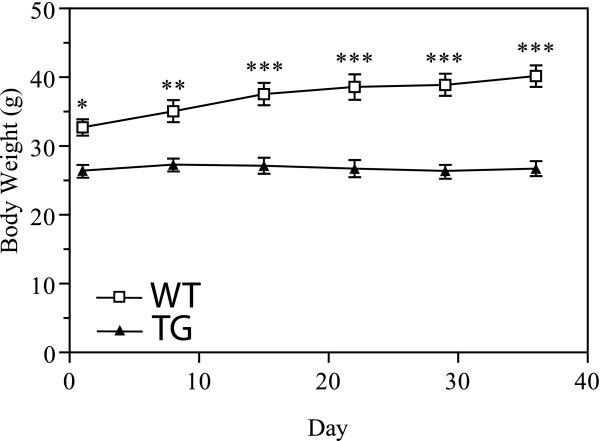
**Weight Gain in Response to a HFD**. TG males failed to increase body weight on a HFD. Weekly body weight measurements for WT (open squares n = 8) and TG (closed triangles n = 8) males taken over the course of five weeks on the HFD. Results represent the mean ± SEM. The level of significance is shown by asterisks: * *P *< 0.05, ** 0.001 <*P *< 0.01, *** *P *< 0.001.

The HFD challenge began when the mice were 23 weeks old. Although there were no differences between genotypes on the regular chow diet, a clear difference in food intake was observed between the WT and TG males measured during the fourth week of the HFD (WT: 94.6 ± 4.8 mg/gm BW/24 hrs [n = 7] vs TG: 122.2 ± 11 mg/gm BW/24 hrs [n = 6]; *P *= 0.0363). In addition to the TG males being lighter and leaner before the HFD challenge, they also failed to significantly increase their body weight in response to the HFD while WT male body weights increased 22.9% in five weeks (Figure 3). The significant differences in weight gain was evidenced by an interaction between genotype and time detected by ANOVA (*F*_5,45 _= 38.0, *P *< 0.0001). DEXA analysis demonstrated that although fat mass was slightly increased in the hTNFα TG mice following the dietary challenge, they remained markedly leaner than the WT group (Table 4).

#### Regional Adipose Distribution

**Figure 4:**
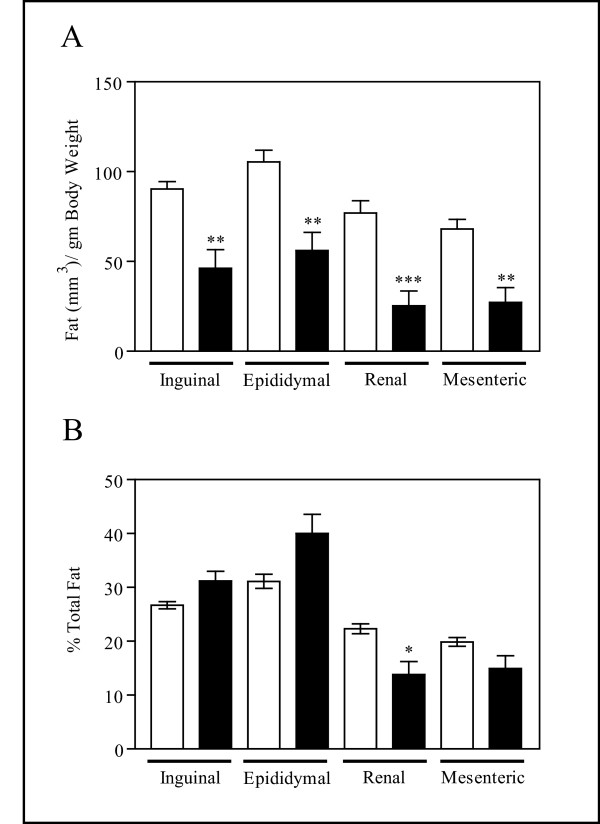
**MicroCT Analysis of Regional Adipose Distribution**. TG males failed to increase regional fat depot volumes following a HFD. (A) The amount of fat in cubic millimeters quantitated by microCT analysis and normalized by body weight is shown for WT males by open bars and TG males by closed bars. The individual depots are shown below the X-axis. (B) The percent of fat distributed in each adipose depot relative to total adipose tissue quantitated by microCT is shown for WT males by open bars and TG males by closed bars. The individual depots are shown below the X-axis. Results represent the mean ± SEM, WT n = 6 and TG n = 7. t indicates P < 0.10, * *P *< 0.05, ** 0.001 <*P *< 0.01, *** *P *< 0.001.

To extend the observation that total adipose tissue was significantly decreased in the TG males, microCT analysis was used to examine individual adipose depots during the fourth week of the HFD experiment. Figure 4A shows the volumes of the inguinal, epididymal, renal and mesenteric fat depots normalized by body weight. Consistent with the results from the DEXA scan (Table 4), the TG males showed significant decreases in all adipose depots examined. To normalize adipose distribution, the sum of all four depots was used. Normalized adipose volumes for each individual depot were expressed as percent of the total fat. The normalized volumes of regional fat depots, shown in Figure 4B, demonstrate that the TG mice had a relative deficit in the percentage of fat stored in the renal depot and a trend towards decreased fat in the mesenteric depot (P < 0.1). Trends reflecting an increase in the percent of fat located in the inguinal and epididymal depots of the TG mice were also observed (P < 0.1). As the renal and mesenteric regions normally exhibit the largest increases in size relative to the other depots in response to a high-fat diet, the differences between the WT and TG mice most likely reflect an impaired ability of the TG males to increase adipose volume in response to the HFD, rather than a direct role of TNFα in fat redistribution.

#### OGTT, Insulin Levels and Insulin Sensitivity

**Figure 5:**
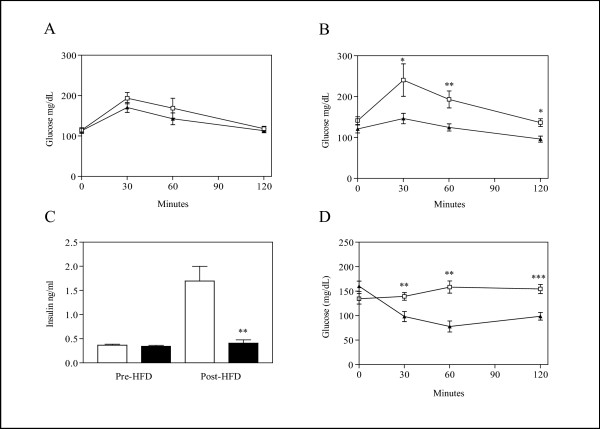
**Glucose Disposal, Insulin Levels and Insulin Sensitivity**. TG mice failed to develop insulin resistance on a HFD. In A, B, and D, WT data is shown as open squares and TG data by closed triangles. (A) OGTT performed on fasted males prior to the start of the HFD showed no significant difference between genotypes (WT n = 7 and TG n = 8). (B) OGTT performed on fasted males following the HFD challenge showed decreased glucose excursion by TG mice (WT n = 7 and TG n = 5). (C) Serum insulin levels in fasted males measured from samples acquired pre and post HFD challenge, WT values are shown by open bars (n = 8) and TG by closed bars (n = 7). (D) Results from the insulin tolerance test performed after the HFD challenge. Insulin levels increased in response to 2 weeks of HFD challenge in WT (n = 7) but not TG (n = 5) mice. All results represent the mean ± SEM. * indicates *P *< 0.05, ** indicates *P *< 0.01 and *** indicates *P *< 0.001 in post-hoc analysis.

Prior to the HFD challenge, baseline measurements of glucose disposal assessed by an OGTT were measured in 21 week old mice and the OGTT was repeated at the end of the HFD challenge at 27 weeks of age. WT and TG groups did not differ in their ability to clear a glucose load before the HFD challenge (Figure 5A) but the TG mice showed a markedly lower glucose excursion compared to the WT group following 6 weeks on the HFD as determined by a main effect of genotype in a two-factor ANOVA (*F*_1,9 _= 10.52, *P *= 0.01) (Figure 5B).

Fasting insulin levels were determined in serum samples collected pre and post HFD challenge at 20 and 27 weeks of age, respectively. There were no detectable differences in fasting insulin levels between the two groups prior to the HFD challenge but following the diet WT insulin levels significantly increased while there was no significant change in insulin levels in the TG males (Figure 5C). A direct measurement of insulin tolerance was performed after 7 weeks on the HFD when the mice were 29 weeks old. The WT mice failed to show a significant decrease in circulating glucose levels at all time points tested in response to an *i.p*. injection of insulin, demonstrating diet-induced insulin-resistance (Figure 5D). In contrast, the TG males were extremely sensitive to insulin with a pronounced reduction in glucose levels at all time points evaluated up to two hours following insulin treatment, resulting in both a main effect of genotype (*F*_1,10 _= 10.48, *P *= 0.009) and a significant interaction between genotype and time (*F*_3,30 _= 13.72, *P *< 0.0001) in the ANOVA analysis (Figure 5D). These results confirmed that the TG mice had higher insulin sensitivity compared to the WT controls following a HFD challenge.

#### Metabolic Rate and Physical Activity

The observation that TG males increased their food intake on the HFD but failed to increase their body weight and fat mass suggested that the metabolic rate and/or activity levels of the TG males were altered. To determine this we assessed the physical activity and metabolic rate of the mice after the sixth week of the HFD, when the mice were 28 weeks old, using indirect calorimetry in the CLAMS (Table 5). We observed that VO_2_, VCO_2_, and heat production normalized to body weight were significantly increased in the TG mice, although RER was not significantly different between the two genotypes. Locomotor activity was markedly suppressed in the hTNFα males even though their metabolic rate was increased. We also assessed the metabolic measures and activity of a separate group of 27 week old TG and WT males maintained on regular chow. These results showed differences between the two groups similar to the differences observed in mice on the HFD (Table 5), ruling out the HFD challenge as the cause for the observed differences in metabolic rate and locomotor activity.

**Table 5:** Metabolic measures and activity measured in the CLAMS

**Parameter**	**WT (n = 8)**	**TG (n = 6)**	**TG Percent of WT**	**WT (n = 7)**	**TG (n = 5)**	**TG Percent of WT**
	
	***HFD***	***HFD***	***HFD***	***RC***	***RC***	***RC***
Oxygen consumption ml/kg/hr	2989 ± 93	3823 ± 110	128***	2770 ± 106	3454 ± 197	125**
Carbon dioxide production ml/kg/hr	2336 ± 114	2932 ± 124	124**	2510 ± 160	2985 ± 220	118*
Heat production Kcal/g	0.014 ± 0.001	0.018 ± 0.001	127***	0.014 ± 0.001	0.017 ± 0.001	123**
Respiratory exchange ratio	0.789 ± 0.015	0.764 ± 0.016	97	0.902 ± 0.036	0.856 ± 0.025	95
Ambulatory activity beam breaks/hr	1220 ± 65	458 ± 102	36**	1839 ± 283	448 ± 72	25***
Vertical activity beam breaks/hr	2124 ± 91	468 ± 133	21***	2550 ± 289	603 ± 166	22***

Values represent the mean ± SEM. The level of significance is shown by asterisks: * *P *< 0.05, ** 0.001 <*P *< 0.01, *** *P *< 0.001. HFD – high fat diet; RC – regular chow; TG percent of WT – measures of TG mice expressed as percent of the measures from the WT group.

### Bone Density and Response to a Low Calcium Dietary Challenge

**Figure 6:**
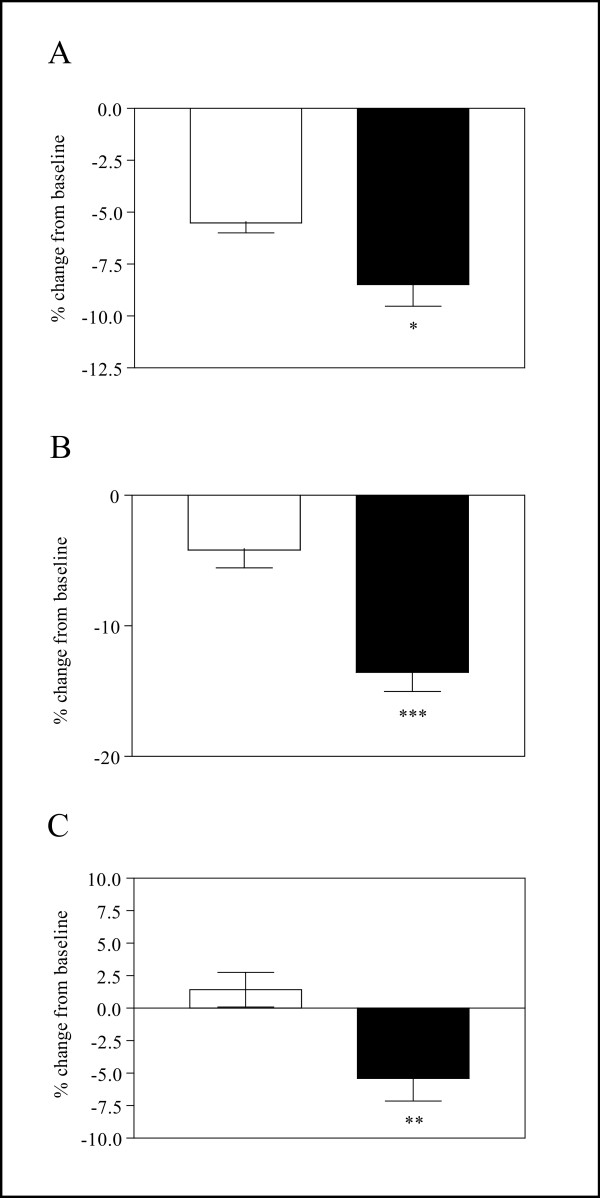
**Response to Dietary Calcium Restriction**. TG females were more susceptible to bone loss in response to dietary calcium restriction. Female mice were analyzed using DEXA prior to and following dietary calcium restriction for 4 weeks. The percent change from baseline for (A) Bone mineral density, (B) Bone mineral content, and (C) Bone area is shown. WT values are shown by open bars (n = 7) and TG (n = 8) by closed bars. Values represent the mean ± SEM. The level of significance is shown by asterisks: * *P *< 0.05, ** 0.001 <*P *< 0.01, *** *P *< 0.001.

DEXA analysis was used to assess bone mineral density (BMD), bone mineral content (BMC) and bone area in the TG (n = 7) and WT (n = 8) mice maintained on regular chow at 22 weeks of age. Significant reductions in BMD (WT: 0.0477 + 0.0007 vs TG: 0.0428 + 0.0005, *P *< 0.001) and BMC (WT: 0.425 + 0.0075 vs TG: 0.378 + 0.0053, *P *< 0.001) were observed in TG males, although bone area was not significantly reduced. In 22-week-old WT (n = 7) and TG (n = 8) females, BMD (WT: 0.0479 + 0.0002 vs TG: 0.0442 + 0.0006, *P *< 0.001) and BMC (WT=: 0.4192 + 0.0052 vs TG: 0.383 + 0.0058, *P *< 0.01) measured in the whole animal were also significantly decreased. In addition, excised femurs and lumbar vertebrae (L1 to L6) from the same females were examined using DEXA. BMC and bone area were significantly reduced in the TG femurs and both BMD and BMC were markedly lower in the excised vertebrae from TG females relative to WT controls (data not shown). In order to further explore this phenotype in females, a low calcium dietary challenge was used. Initially, TG and WT mice were scanned by DEXA to establish baseline values for BMD and BMC. The mice were then fed a diet containing only 1.25% of the calcium available in regular chow for four weeks. A second DEXA scan was performed on 27 week old females and the changes in BMD, BMC and bone area were compared. The BMD and BMC of TG females were significantly lower than the WT controls prior to the restriction in dietary calcium as shown above and these differences were exacerbated in response to the diet (Figure 6A–C). TG females were more susceptible to loss of BMD, BMC and bone area compared to WT mice when measured as the percent change from baseline.

### Male sexual behavior and fertility

Male sexual behavior was tested at 17 weeks of age in which the number of males that mounted receptive females, performed intromissions and ejaculated were compared between genotypes. Significantly fewer TG than WT males mounted females during the 45 min test and the number of TG males that performed intromissions was also significantly lower compared to the controls (Table 6). The number of mounts and intromissions per performing male, as well as latencies to the first mount and intromission were similar in the TG and WT groups (data not shown).

**Table 6:** Number of hTNFα and WT mice performing male sexual behaviors

**Parameter**	**TG**		**WT**		***P*-value**
		
	***Present ***	***Absent ***	***Present***	***Absent***	
Mounting Behavior	7	5	12	0	0.012
Intromission	2	10	9	3	0.041
Induced erection (1)	7	8	14	1	0.005
Induced erection (2)	9	3	12	0	0.064
Sired pregnancies	4	8	9	3	0.041

Penile erection was tested in two separate experiments comparing TG and WT males in the induced erection test. In the first experiment significantly fewer TG males displayed an erection compared to WTs (Chi-square, P = 0.005). In the second study there was still a difference but it did not reach statistical significance (Chi-square, P = 0.06, Table 6).

A significantly smaller number of the TG males sired pregnancies after one week with CD1 females (Table 6). All pregnant females gave birth to live litters. There was no effect of the transgene on the litter size detected at this age (12.75 ± 0.9, n = 4, TG; 12.67 ± 0.4, n = 9, WT). The analysis of hematoxylin/eosin stained sections of the prostate, epididymis, seminal vesicles and penises, in addition to examination of PAS/hematoxylin stained sections of testes from two TG and two WT mice, did not reveal any differences due to the presence of the transgene (data not shown).

## Discussion

The TG mice reported here develop progressive arthritis [21] similar to previously described transgenic lines generated with this construct [16] but do so at a later age, consistent with the lower levels of circulating TNFα than in the lines reported earlier [18,23] and more closely resembling the progression of RA in humans. We generated several lines of transgenic mice with this construct and although all of the lines had an arthritis phenotype we chose this line for a more thorough characterization because of its slower onset of the arthritis phenotype. In the generation of transgenic mice the integration site can be a factor in observed phenotypes because there is no control over the copy-number of transgenes that integrate and possible direct or indirect influences from the integration (unintended mutations of other genes or cis-genetic effects influencing expression). Thus, although the expression level in this line has utility as a model for human RA progression, a direct comparison between multiple lines with similar, low levels of expression is required to formally prove that the phenotypes observed here are due to the deregulated TNFα expression. Nonetheless, in addition to RA found in this line as well as previously published lines, the reduced body weight, increased metabolic rate and restricted motor activity correlate well with similar findings described in humans with rheumatoid cachexia [6]. Bone loss, which has also been previously described [23] was documented in the line described here. Lastly, the male TG mice have some sexual behavior dysfunctions that could be attributable to moderate disease progression of RA but the low scores for induced erection tests suggest that the sexual behavior phenotypes may be due to multiple causes. Our approach of investigating the pleiotropic actions of TNFα by phenotyping the same mice in multiple *in vivo *assays confirms the results of many studies conducted on different lines of transgenic mice. Further, our results are consistent with an interpretation that many of the effects of deregulated TNFα expression are not directly a result of the development of RA.

Mice expressing the hTNFα transgene driven by its endogenous promoter but containing the β-globin 3' flanking region in place of hTNFα 3'sequences have previously been described as a model for progressive arthritis [5,16]. The replacement of the endogenous 3' UTR of the hTNFα gene with β-globin 3' sequences renders the gene non-inducible in macrophages and also stabilizes the mRNA in hematopoetic and stromal cells, leading to constitutive expression of the transgene [16,24]. Consistent with these studies, we demonstrated here that LPS treatment did not induce expression of the transgene but inducibility of the endogenous gene remained intact, as expected. Several lines have been generated using this modified construct [5] and common observations include progressive arthritis and a wasting phenotype. In the most extensively studied line (TG197) both phenotypes are manifest by 4 to 6 weeks of age and early mortality is common [16]. This rapid onset of arthritis and wasting may be attributable to the relatively high circulating concentrations of hTNFα in this line [18,23]. The hTNFα transgenic line studied here was generated using the same construct as line TG197 but expresses significantly lower levels of circulating hTNFα and has a later onset of arthritis in addition to longer life spans, which more closely models the disease progression in humans. Therefore, this mouse line may provide a more appropriate model for the pathophysiological effect of increased levels of TNFα. One caveat that should be pointed out in this model is that our transgene is likely expressed principally in stromal cells, since it was previously shown that deletion of the 3'-region of the human TNFα gene resulted in the loss of macrophage-specific TNFα expression [16]. This pattern of expression may not be consistent with all clinical pathologies associated with increased TNFα levels.

Humans and mice both possess two receptors for TNFα, TNFR1A (p55) and TNFR1B (p75) [25]. The generation of knockout mice for both TNFR1A and TNFR1B, have identified specific roles for these two receptors. For example, TNFR1B is required for TNF-induced T-cell toxicity and tissue necrosis [26] but is not required for TNFα-mediated lipolysis or inhibition of insulin-stimulated glucose transport [27]. TNFR1A is thought to be the primary signaling receptor on most cell types involved in resistance to bacterial infections and the lethal response to endotoxins [15]. TNFR1A also inhibits the differentiation of adipocytes, negatively affects lipid accumulation and insulin sensitivity in mature adipocytes [27], and regulates leptin production [28]. Additionally, TNFR1A mediates negative effects on bone density [29], primarily by increasing the number of osteoclast progenitors and stimulating their differentiation and activity, but also by suppressing the differentiation of osteoblasts. A species specificity was demonstrated since human TNFα binds with high affinity only to murine TNFR1A [25] thus, phenotypes identified in this study should mainly reflect processes mediated by TNFR1A.

The hTNFα TG mice in this study demonstrated sexual dimorphisms in a number of physiological measures despite similar levels of circulating hTNFα. The female TG mice appeared less affected by the expression of the transgene than the males. For example, significant differences between the sexes were observed in the Irwin test, where females were not phenotypically distinct from the WT controls but age-matched males suffered reduced grip strength and wire maneuver, likely as a result of the advanced arthritis in the males (Table 3). TG males also displayed a more pronounced decrease in body weight and soft tissue composition than did the females (Figure 2; Table 4). While the basis for the sexual dimorphisms in this TG line was not investigated, previous studies have demonstrated that estrogen exerts a protective effect against chronic exposure to hTNFα [30]. Significantly fewer TNFR1A receptors in heart muscle of female mice have also been reported although the liver and peripheral white blood cells display equivalent numbers of TNFR1A receptors [20]. It is unknown whether the density of this receptor in females is also lower in other tissue types. By 14 weeks of age many male TG mice had already developed paw swelling and eritema consistent with moderate arthritis, which is likely responsible for the observed phenotypes in the neurological tests in Irwin that were specific to the males (see [21] under "phenotype" for supplemental data). There were no visible signs of arthritis in age-matched females.

The dramatic decrease in BMD and BMC in the TG mice of both sexes (see Results), and the accelerated bone loss observed on a low calcium diet in the TG females (Figure 6) are consistent with the established role of TNFα in bone resorption. A significant loss of BMD is also characteristic of advanced RA [29,31-33].

Numerous studies have investigated the ability of TNFα to modulate leptin expression and secretion. Acute administration of LPS or relatively high levels of TNFα increase leptin in both humans and mice [34-36]. However, low levels of leptin have been measured in patients with both chronic inflammatory conditions and cachexia [37-40], raising the possibility that repression of leptin reflects biological compensation in response to these conditions. In addition to leptin's well established roles in inhibiting food intake and increasing energy expenditure there is also a growing body of evidence indicating that leptin can regulate bone density, potentially through a central hypothalamic response [41-43]. Thus, the results from our comprehensive screen could be interpreted as a down-regulation of leptin in an effort to reduce metabolic rate, bone loss, and/or depletion of adipose stores. Alternatively, suppression of circulating leptin may result from chronic stimulation of receptor signaling pathways shared with TNFα receptors.

Contradictory results describing effects of TNFα on glucose tolerance and insulin sensitivity have been previously discussed [44,45]. In the TG mice described here low circulating levels of TNFα did not significantly affect glucose disposal in mice fed regular chow. However, the TG mice did not develop impairments in insulin sensitivity and glucose tolerance like the WT mice did when fed the HFD for 7 weeks. These findings are likely the result of the blunted weight response of the TG mice to the HFD as well as their maintenance of normal insulin levels.

The reduced body weights, altered soft tissue composition, and increased metabolic rate in the male TG mice presented here are hallmarks of the cachectic state in humans. Cachexia caused by arthritis, cancer and AIDS have all been correlated with elevated TNFα levels [46]. Common observations include catabolic effects on body mass with lean tissue, particularly skeletal muscle, more affected than adipose stores [46-48]. An increased resting metabolic rate is a frequent finding, and anorexia is found in many but not all cases. In mice, a lethal wasting phenotype has been reported in animals expressing hTNFα under the control of T-cell specific regulatory sequences [17] and decreased body weight was observed in previously reported lines harboring the transgenic construct used in this study [16]. In the phenotypic analysis of the hTNFα TG mice presented here, the metabolic rate was significantly elevated regardless of diet and both adipose tissue and lean mass were significantly decreased. However, our results indicate that in this model, fat mass is affected more than lean mass. In addition, the TG mice did not display anorexic behavior since they consumed comparable amounts of regular chow and were even hyperphagic on the high-fat diet. However, the profoundly decreased physical activity of the TG mice is consistent with "sickness behavior" [49,50]. Despite the obvious alterations in metabolic rate, body composition, and body weight, these TG mice do not demonstrate excessively early mortality. Male TG mice were maintained for over a year without obvious differences in survival compared to WT mice (unpublished observation). These data suggest that the cachexia seen in RA may not be directly caused by TNFα but the cytokine may be a contributing factor and is responsible for many of the other metabolic and physiological symptoms observed in rheumatoid cachexia as well as in our mouse model.

Although some of the phenotypes in male sexual behavior in the hTNFα TG mice observed in this study were likely due to arthritic-like lesions of the limbs and the compromised health status of the animals we also showed that the erectile function of the TG mice was decreased, which is likely an additional factor contributing to the impairment in male sexual behaviors. It has been proposed that iNOS-mediated chronic NO overproduction in the penile tissue could be a factor in declining erectile function associated with aging and in Peyronie's disease in humans and rat models [51,52]. A number of studies suggest that TNFα activates iNOS mediated NO production in endothelial and smooth muscle cells of blood vessels [53,54]. Sustained up-regulation of iNOS may result in chronic overproduction of NO in the penile tissue of the hTNFα TG mice and be eventually responsible for the impaired erectile function of the mice observed in this study. The reduced sexual activity of the TG mice likely contributed to the decreased number of pregnancies sired by the TG males compared to the WT controls observed in this study. In addition, chronic exposure to the circulating hTNFα might also affect the germ cell development and sperm maturation in the TG males, as multiple effects of TNFα on the biology of the testicular Sertoli cells were reported in the literature [55-58].

Our results have confirmed many of the previously demonstrated traits associated with constitutive expression of TNFα. In addition, this study revealed several novel physiological effects of TNFα such as the effects of TNFα on induced erection and a sexual dimorphism of many of the observed phenotypes. This study demonstrates a number of divergent phenotypes in the same TG line and even the same cohort of subjects, illustrating the pleiotropic role of TNFα and the cross-physiological effects of cytokines such as TNFα, which regulate or modulate immunological, metabolic and nervous system processes.

## Conclusion

In summary, our phenotypic analysis of the hTNFα TG line demonstrates the utility of this mouse line as a model for multiple human diseases in which chronically elevated TNFα levels are present including its established use in studies of RA. Chronic low levels of circulating hTNFα resulted in defects in bone homeostasis, a hypermetabolic state characterized by low body weight and hyperphagia but coincident with low motor activity, depletion of adipose tissue and lean mass, lack of a weight increase with a HFD challenge, inappropriately low leptin levels and impaired male sexual health. In addition, these studies also revealed that a significant level of sexual dimorphism exists with respect to TNFα 's role in the progression and severity of several biologically relevant phenotypes.

## Authors' contributions

MDH drafted and revised the manuscript and served as the corresponding author; BKJ drafted the manuscript, prepared tables, contributed to the study design and interpretation of results; AS contributed to the study design and interpretation of results; HSH contributed to the study design and interpretation of results; A-CT contributed to the study design and interpretation of results; MMB developed and conducted assays, performed statistical analyses and made figures, AC conducted assays and statistical analyses; BL-W conducted clinical chemistry assays and statistical analyses; BS conducted in vivo assays and statistical analyses; CG managed animal husbandry and health; HS conducted assays and statistical; EA conducted in vivo assays and statistical analyses; JW conducted in vivo assays and statistical analyses; MV conducted in vitro assays and statistical analyses; MT conducted in vivo assays and statistical analyses; RHF made the construct for transgenic mice and genotyped mice; WC conducted in vivo assays and statistical analyses; YR conducted in vivo assays and statistical analyses; DSG created transgenic animal line, identified arthritic phenotype, reviewed and approved experimental plan, and reviewed manuscript for final approval; OB designed experimental plan, drafted manuscript, and tables and reviewed manuscript for final approval.

All authors read and approved the final manuscript.
